# The connotations of Value-Based Healthcare: interpretive perspectives and differences in perceptions

**DOI:** 10.3389/fpubh.2026.1866891

**Published:** 2026-06-17

**Authors:** Wenneng Jiang, Shuangshuang Lu, Cuiying Xie, Qingqing Shu, Guohui Song

**Affiliations:** School of Public Health and Management, Guangxi University of Chinese Medicine, Nanning, China

**Keywords:** cognitive differences, connotation of healthcare, perspectives of understanding, public welfare orientation, Value-Based Healthcare (VBHC)

## Abstract

**Objective:**

This study aims to elucidate the connotations of Value-Based Healthcare (VBHC), correct misconceptions, and explore perceptual differences among distinct groups.

**Methods:**

An analytical framework for VBHC connotations was first constructed from five perspectives: subject-object and subjective-objective relations, the individual and the collective, the refined value equation, conceptual categories and scope, and value implications. A questionnaire was then developed, and statistical analyses, including analysis of variance (ANOVA) and chi-square tests, were conducted on the collected survey data.

**Results:**

Significant differences existed between older adults and other age groups regarding the public welfare orientation, equity, and cost perceptions. Perceptions of “patient-centeredness,” subjective utility, and marginal value varied significantly by education, region, and urban–rural residency. Compared to other cohorts, older adults prioritized health outcomes, and middle-aged to older groups focused on functional recovery, whereas survival duration was not a primary concern. Individuals with graduate degrees, the public, patients, and urban residents prioritized health outcomes; conversely, other groups balanced health outcomes and costs, though none prioritized costs alone. Healthcare leaders prioritized hospital expenses, whereas others prioritized patients’ financial burdens. Although functional recovery was universally favored, urban residents valued survival duration, whereas county and township populations emphasized the likelihood of disease recurrence and recovery sustainability.

**Conclusion:**

(1) Overall, respondents exhibited weak cost awareness; rather than prioritizing health outcomes or costs individually, they favored a balance between health outcomes and costs and prioritized functional recovery over survival duration. (2) Older adults and eastern populations leaned toward the individual, the public welfare orientation, and efficiency, whereas other age cohorts and western populations favored the collective, equity, and the public interest. Central populations focused on the collective but attempted to reconcile equity and efficiency, embodying a unique, moderate value paradigm. (3) Urban populations interpreted “patient-centeredness” from a multi-stakeholder perspective, emphasizing objective health outcomes and survival duration, whereas county/township populations more readily accepted subjective utility and diminishing marginal value, considering costs more frequently.

## 1 Introduction

Since its introduction by Porter and Teisberg (1), Value-Based Healthcare (VBHC) has emerged as a guiding framework for healthcare reforms worldwide, injecting a powerful impetus and boundless vitality into China’s new round of healthcare reform. Although China’s reform is not explicitly labeled as VBHC, the philosophy of VBHC is deeply embedded within numerous policy documents. Consequently, the reform has yielded substantial achievements, most prominent of which is a heightened emphasis on cost control. By systematically reducing healthcare costs, the reform aims to serve the public interest, ensuring that the broader population can share in the dividends of healthcare progress. This trajectory is evidenced by the 2025 China Health Statistical Yearbook, which reports that in 2024, the average medical expense per hospital inpatient decreased by 10.29%, even as the total number of hospital admissions and patient visits grew by 3.93 and 5.70%, respectively.

Despite these achievements, deviations persist in the formulation of specific policy measures, the selection of policy instruments, and their implementation, generating new problems and contradictions. For instance, on the one hand, national basic medical insurance funds have accumulated massive surpluses, reaching a cumulative balance of approximately RMB 3.863 trillion by the end of 2024 (2). On the other hand, total medical and pharmaceutical spending among enrollees in both employee and resident medical insurance schemes has continued to rise rapidly. The gap between these total expenses and the payouts from the national basic medical insurance fund (a positive difference that essentially represents the out-of-pocket cash expenditures of insured individuals) remains substantial, accounting for a large proportion—between 25 and 40%—of the fund’s total expenditures (3). In addition, practices such as split hospitalizations, patient dumping, and hospital closures are increasingly prevalent. Furthermore, centralized volume-based procurement (CVBP) has led to a drastic reduction in the proportion of originator drugs, raising concerns about clinical effectiveness—as highlighted by experts such as Director Zheng Minhua, a member of the Shanghai Municipal Committee of the Chinese People’s Political Consultative Conference (4).

What accounts for these two divergent phenomena, particularly the newly emerging problems and tensions? This paper argues that the fundamental reason lies in deviations in perceptions regarding the connotations of Value-Based Healthcare (VBHC), which subsequently skew policy design. For example, should healthcare system reform prioritize cost control or the improvement of treatment outcomes? Professor Li (5, 6), a staunch advocate of free healthcare, posits that controlling medical costs enables hospitals to serve the public interest, thereby achieving equitable and accessible healthcare. Professor Liu (7), despite leaning toward a market-oriented approach, also focuses primarily on cost control. He advocates reducing healthcare costs by channeling resources to lower-level facilities, implementing a tiered diagnosis and treatment system, and enhancing primary healthcare service capacity. However, recent studies by his research team (8, 9) found that separating drug sales from medical services has had a limited impact on healthcare costs and quality, with negligible long-term effects; furthermore, while the “first contact at primary care” policy significantly reduced the initial visit choices and medical expenses for the older adults, it did not alter the initial visit choices or medical expenses of the non-retired population. Leusder et al. (10) argue that cost is the key to pursuing value and advocate for enhanced cost measurement research. In their review of existing studies on healthcare costs, they found that 51.2% of the 215 cost measurement studies conducted within a VBHC context measured hospital costs. They conclude that cost information can facilitate VBHC and identify Time-Driven Activity-Based Costing (TDABC) as the optimal method.

Regarding the definition of Value-Based Healthcare (VBHC), Michael Porter asserts that value must be defined around the customer, which means creating value for the patient. The ultimate value of health services must be judged by measuring both outcomes and costs, concisely defining value as the ratio of outcomes to costs (11). Within the academic community, VBHC is often characterized as the pursuit of healthcare with the highest cost-effectiveness (12). The VBHC framework proposed by Porter has sparked intense debate. One perspective argues that the “value” Porter refers to is defined relative to healthcare providers or principal payers and is thus primarily an economic value (13). This approach preferentially employs cost-effectiveness analysis, effectively equating “value” with “economic value” (14). Consequently, Porter and Lee (15) later broadened the connotations of VBHC to include not only economic value but also social value, establishing VBHC as a multidimensional concept. Subsequently, scholars have advanced research along this multidimensional trajectory. The European Commission’s Expert Panel on Effective Ways of Investing in Health (EXPH) proposed four pillars of value creation in health systems: health, equity, person-centeredness, and social participation, which essentially correspond to technical value, allocative value, personal value, and societal value. Zhang et al. (16) reviewed relevant studies and categorized value attributes into nine domains: health gains from health technologies, quality of evidence, cost-effectiveness, innovativeness, disease burden, unmet needs, affordability, ethics and equity, and social impact. They concluded that the involvement of patients or the public in the development of existing frameworks is limited or entirely absent. Smith et al. (17) examined value creation in health systems, defining health system value as its contribution to societal well-being, and constructed a five-dimensional value framework comprising health improvement, healthcare responsiveness, financial protection, efficiency, and equity. Evidently, multidimensional research has substantially expanded the connotations of value. In light of this, Hu (18, 19) summarized the value of healthcare services into two dimensions: micro-level value and macro-level value. Precisely because of this conceptual expansion, some scholars have investigated whether evaluation metrics based on VBHC truly reflect the principle of patient-centeredness. For instance, Kidanemariam, et al. (20) reviewed related studies and found that the most frequently used research indicators in VBHC are not patient-centered; rather, they appear to be healthcare quality metrics defined from the perspectives of providers, institutions, or payers.

Another line of inquiry follows Porter and Teisberg’s (1) early, concise formulation of value by exploring the interplay between patient outcomes and healthcare costs. For instance, value-based healthcare (VBHC) has been conceptualized as the value created for patients, serving as a composite measure that integrates the utilization, costs, and expenditures of comprehensive medical services. Similarly, Jin et al. (21) propose that the quantitative measurement of VBHC is obtained by dividing patient outcomes or service quality by the total expenses incurred. Furthermore, some scholars note that value enhancement manifests primarily in two scenarios: either clinical effectiveness improves while costs decrease or remain unchanged, or effectiveness holds steady while costs are reduced (22). In addition, other researchers contend that the precise connotation of value remains ambiguous; beyond the health outcomes achieved per unit of expenditure, factors such as patient experience, loyalty, and equity are also regarded as critical components of value, yet a unified definition across these dimensions has not been established (23). This ultimately raises a fundamental question: what constitutes true “value” in healthcare? Is it merely a matter of minimizing medical costs and ensuring that patient satisfaction is “good enough,” or must it be anchored in clinically appropriate care? Current literature still lacks a comprehensive evaluation that systematically integrates these theoretical and practical dimensions (24).

Furthermore, it is generally acknowledged that perceptions of Value-Based Healthcare (VBHC) vary significantly across different populations. A report by the Institute of Medicine (25) pointed out that the meaning of “value” differs markedly among stakeholders: healthcare providers often define value based on the appropriateness, effectiveness, and evidence base of an intervention, whereas patients are more likely to understand the value of a health technology from the perspectives of service accessibility, equity, and quality improvement. Research by Smith et al. (17) posits that the concept of value diverges among different actors within the health system. Similarly, Wolf et al. (26) argue that the difficulty in employing the concept of “value” lies in the fact that different subjects understand it differently across various contexts and medical events; through qualitative research, they found a distinct perceptual gap between clinical and non-clinical personnel, with clinicians primarily focusing on enhancing treatment efficacy and improving medical outcomes while paying insufficient attention to costs and the outcomes that patients truly value. Evidently, substantial controversies persist regarding the connotations of VBHC. Amid these theoretical debates, the definition of value has gradually deviated from its “patient-centered” trajectory, skewing instead toward the perspectives of the general public or medical professionals. To clarify these controversies, accurately grasp the scientific essence of VBHC, and specifically correct the perceptual bias of overemphasizing cost control, this paper contends that it is necessary to construct an analytical framework for the connotations of VBHC grounded in the fundamental principle of patient-centeredness. By expanding the scope of the surveyed populations beyond patients and medical professionals to include the general public and leaders of relevant institutions, this study conducts an empirical analysis. The aim is to discern current trends and differences in how people perceive the connotations of VBHC, analyze the underlying reasons, and provide reference points for perfecting China’s healthcare system reform.

## 2 An analytical framework for interpreting the connotations of value-based healthcare

Following the fundamental principle of patient-centeredness, this paper constructs an analytical framework for the connotations of Value-Based Healthcare (VBHC) from the following five perspectives.

### 2.1 The relational category of VBHC: subject–object relations or subjective–objective relations?

Philosophically, the connotation of value is primarily explained through frameworks such as the “attribute theory,” “entity theory,” “relational theory,” and “teleological theory.” Among these, the “relational theory” posits that value is the product of the interaction between a subject and an object (27). Value represents a relationship of significance—essentially a relationship of interests—between the subject and the object. While value relationships are objective, value conceptions are subjective (28).

When interpreting the connotation of Value-Based Healthcare (VBHC) through the lens of relational theory, two distinct perspectives emerge. The first perspective examines the connotation of VBHC based on the relationship between the subject and the object. Healthcare services involve a multitude of stakeholders, including patients, healthcare institutions, medical professionals, administrative agencies, governments, the public, pharmaceutical companies, and insurance firms. All these actors interact in pursuit of maximizing their own interests. However, as Porter asserts, value should always be “defined around the customer.” In an ideal health system, the value created for patients should determine the rewards for all participating parties (11). VBHC advocates for patient-centeredness precisely because patients’ preferences, needs, and values can mediate the differences—or even conflicts—among the preferences, needs, and values of other stakeholders. This mediation enables all parties to maximize their respective interests, thereby optimizing the overall value of healthcare. No other stakeholder possesses this unique mediating function. The second perspective examines the connotation of VBHC based on the relationship between subjectivity and objectivity. Objectivity is manifested in the usefulness of healthcare services in meeting patients’ needs for restoring or maintaining biological bodily functions. Subjectivity, on the other hand, is primarily reflected in the restoration or maintenance of patients’ normal psychological and social functions (which inherently contains some objective elements as well). For example, contemporary health assessments or Health Technology Assessments (HTA) evaluate dimensions such as efficacy (effectiveness), safety, economic viability (costs, expenses, effects, and benefits), and social adaptability or impact (social, ethical, moral, and legal aspects) (29). Clearly, this constitutes a comprehensive assessment of “usefulness” that integrates both subjective and objective components. Another example is health economic evaluation. According to mainstream economic theory, such evaluation is inherently an assessment of utility, making its subjectivity even more pronounced. Specifically, it necessitates evaluating the allocative and productive efficiency of scarce healthcare resources in alignment with the health demands of patients and the broader public. As noted in the literature, “Value is not something inherent in the object itself; rather, it arises from the relationship between the characteristics of an object and a person’s desires or needs. Value is subjective” (30).

### 2.2 “The patient” in “patient-centeredness”: individual or collective?

At this juncture, it is essential to clarify the critical concept of “public welfare orientation,” as it is frequently conflated with “public interest.” Ren (31) examined the thoughts of philosophers like Habermas alongside the etymological origins of the term “public” to clarify two analytical dimensions of its definition. The first dimension concerns the “public” in relation to the “private,” delineating the boundary of the public sphere when contrasted with private life. The second dimension focuses on the public sphere itself, involving its substantive and formal meanings, the latter of which corresponds to the contemporary concept of “publicity.” Wang and Chen (32) noted that “public” originates from two ancient Greek terms. One term emphasizes an individual’s capacity to transcend self-interest to understand and consider the interests of others. The other term describes a state of mutual care and concern among people in their work and social interactions. Similarly, Liu and Guo (33) distinguished between the concepts of “public welfare” and “social welfare.” Public welfare embodies humanitarian, humanistic, and people-centered values, whereas social welfare reflects the responsibilities and functions of the state. Lei (34) also recognized the distinction between a public welfare orientation and the public interest. Consequently, he categorized the public welfare orientation of hospitals into “natural” and “derived” forms. The natural public welfare orientation represents altruism and philanthropy. Conversely, the derived public welfare orientation emphasizes serving the public interest, while still retaining certain public welfare elements such as providing free medical services.

Based on this conceptual genealogy, interpreting the “patient” as either an individual or a collective entity leads to divergent understandings of the connotation of Value-Based Healthcare (VBHC). This paper contends that a public welfare orientation is fundamentally anchored in altruism. It refers to selflessness, philanthropy, and standing in opposition to private interests. In contrast, the public interest is defined in relation to individual interests, representing the welfare of the majority. From the perspective of a public welfare orientation, physicians and hospitals must embody the spirit of healing the wounded and rescuing the dying, without pursuing private gain. From the perspective of the public interest, however, medical professionals and institutions may acquire personal benefits, provided they do not infringe upon the public interest. When the “patient” is understood individually, VBHC predominantly assumes an “efficiency” orientation. The personal value achieved under this model may fulfill the requirements of a public welfare orientation, yet it does not necessarily align with the principles of the public interest. Conversely, it might serve the public interest without possessing a genuine public welfare character. When the “patient” is understood collectively, it reflects collective value and the public interest. In this scenario, VBHC gravitates toward an “equity” orientation, necessitating the balanced allocation of healthcare resources.

Furthermore, one major significance of distinguishing between individual and collective understandings of the “patient” lies in navigating the tension between efficiency and equity. As discussed by Moriates et al. (35), an insurmountable tension exists between what is best for an individual patient (beneficence) and what is best for society as a whole (justice). When the patient’s right to choose diverges from societal interests, clinical and policy decisions become substantially more challenging (35). For example, centralized volume-based procurement (CVBP)—which exchanges volume for lower prices—effectively reduces costs and makes medical care affordable for ordinary citizens. However, it simultaneously drastically reduces the proportion of originator drugs on the procurement list, which can compromise clinical effectiveness. The second major significance is that this distinction clarifies how different stakeholders perceive the “patient.” Whether the “patient” is viewed through an individual or a collective lens depends heavily on the stakeholders’ respective interests and values. Even when sharing the same lens, the specific connotation can vary. For instance, a large-scale survey of American physicians revealed their decision-making priorities when determining a treatment course: 54% considered societal costs, 78% prioritized the patient’s best interests regardless of high prices, and 85% favored allocating these expensive medical resources to patients in more acute need.

### 2.3 The VBHC formula: outcome primacy or cost primacy?

Porter summarizes the connotation of Value-Based Healthcare (VBHC) in a concise formula: Value = health outcomes/costs. This formula can be interpreted from at least three perspectives.

First, the formula implies simultaneously improving health outcomes while reducing costs. As one widely cited formulation puts it, VBHC aims to achieve better health outcomes, quality, and patient safety at lower cost (36). In the long run, with continuous technological progress and diffusion, this ideal state may be attainable; in the short run, however, trade-offs are often unavoidable. Some studies argue that in value-based purchasing of healthcare services by health insurance, no single payment method can simultaneously achieve cost containment, quality assurance, and efficiency improvement (37). From the standpoint of an individual patient, real-world needs are immediate rather than deferred. Patients typically cannot wait for a future point at which technological advances make it possible to deliver both better outcomes and lower costs.

Second, the formula implies holding health outcomes constant while reducing costs. In specific healthcare transactions, if no better approach is available to improve health outcomes, VBHC may be operationalized as minimizing costs. From a dynamic and macro-level perspective, however, such a mindset may steer healthcare providers, and governments in particular, toward cost cutting while under-emphasizing health outcomes. This is because health outcomes, particularly macro-level health outcomes related to the public interest, are difficult to compare or calculate in any straightforward way. Porter therefore repeatedly cautions that cost control without attention to health outcomes is dangerous and can backfire, creating only the illusion of saving money while potentially undermining the effectiveness of health services (11).

Third, the formula implies holding costs constant while improving health outcomes. For instance, Cutler, the architect of the Obama administration’s healthcare reform plan, argues that the true aim of healthcare reform is to create “value,” understood in his discussion primarily in terms of healthcare quality, rather than merely to control costs. Through valuation studies, he further notes that a fundamental driver of cost growth is the increasing sophistication of life-saving capabilities (38). Under this interpretation, VBHC does not equate to low spending per se, but rather to higher value at the same level of spending (37). This VBHC mindset is particularly relevant to the formulation of macro-level health policy, as it guides healthcare organizations, and governments in particular, to focus on healthcare quality and health outcomes.

It is important to guard against conflating the second and third perspectives. Mathematically, “maximizing health outcomes under a fixed cost” is equivalent to “minimizing costs for a given health outcome.” From the perspective of linguistic logic, however, their connotations differ: the former focuses on improving health outcomes, whereas the latter focuses on reducing costs. Porter’s connotation of value aligns with the former rather than the latter.

### 2.4 The scope of VBHC: micro-level or macro-level?

This can be understood through both the connotation and denotation of Value-Based Healthcare (VBHC), encompassing both micro-level (or narrow) and macro-level (or broad) dimensions. Furthermore, within certain boundaries, the micro-level and macro-level dimensions conflict with each other.

First, from the perspective of connotation, the scope of VBHC exhibits a clear trend toward enrichment. This enrichment manifests at the individual patient level as narrow value evolves and expands into broader value. For instance, a patient’s needs when receiving hospital treatment dynamically shift among health outcomes, costs, and medical experiences. Patients evaluate the magnitude of healthcare value by comparing health outcomes against their personal financial outlays. Simultaneously, they factor in their healthcare experience. Notably, when health outcomes are less pronounced, patients’ desire for a high-quality medical experience becomes even more intense. Second, at the collective patient level, this enrichment is characterized by the expansion of micro-level value into macro-level value, alongside the tensions between them. At the micro level, value-centered healthcare is articulated as patient-centered care (39). Conversely, at the macro level, it encompasses patients as well as all participants within the healthcare ecosystem. These include family members, physicians, payers, suppliers, and other relevant stakeholders (39). Optimizing value for all stakeholders across the system requires extending the measurement of value to the societal level (39). Macro-level value entails providing services characterized by quality, safety, effectiveness, timeliness, equity, and efficiency (18). However, conflicts frequently arise between micro-level and macro-level values. For example, health outcomes at the micro level (representing efficiency) often stand in direct opposition to healthcare accessibility at the macro level (representing equity).

Second, from the perspective of denotation, the scope of VBHC continuously expands from the micro level to the macro level. Micro-level value refers to value understood from a specific, localized aspect. In contrast, macro-level value pertains to value creation conceptualized across a broader region, the entire nation, or the whole healthcare system. It can also span a prolonged timeframe, such as the full life cycle. Tensions also exist between these two dimensions. For instance, isolated clinical acts, such as a specific diagnosis or a surgical procedure, can successfully create value for a patient locally. However, when evaluated against the systemic nature of the living organism or full-life-cycle health, the outcome may be entirely different. If systemic initiatives—such as the implementation of a tiered diagnosis and treatment system, the reform of county-level medical consortia, or the restructuring of differential medical insurance reimbursement rates—merely retain patients at appropriate facilities and boost county-level consultation rates but result in poor health outcomes or even delay optimal treatment timing, they may possess only institutional macro-level value, while lacking micro-level value and, consequently, substantive macro-level value.

### 2.5 The significance of VBHC: incremental or stock?

Measuring the value of healthcare services relative to a baseline value status reveals the relationship between stock value and incremental value. Both concepts, along with the more widely used term “marginal utility,” are economic constructs and share very similar meanings. However, this paper deliberately avoids the concept of marginal utility. This choice stems from our contention that the utility-based conception of value is inherently subjective rather than objective, whereas the value of healthcare services can encompass both objective and subjective dimensions. Objectivity is demonstrated by the fact that physiology and pathology provide objective clinical indicators to evaluate the value of healthcare services. Therefore, as explained earlier from the first perspective of the “subjective-objective relationship,” medical value is both objective (manifested as usefulness) and subjective (manifested as utility). The core connotation of value must be understood through the interplay between these two dimensions. Furthermore, the law of diminishing marginal utility in mainstream economics dictates a strict downward trajectory, which fails to accurately capture the specific usefulness and effectiveness of healthcare services.

First, incremental value. This refers to the value-added effect that healthcare services generate for patients. Fundamentally, Value-Based Healthcare (VBHC) operates as value in an incremental sense, representing a type of marginal value. This can manifest as a value increase for individual patients, or for patient collectives and the public. It can occur within micro-level domains or expand into macro-level areas. Furthermore, it can represent a direct value enhancement for patients, or an indirect value gain. In terms of health outcomes and costs, it can manifest as increased health outcomes at a fixed cost, reduced costs for a given level of health outcomes, or simultaneously improving health outcomes while lowering costs. However, this ultimately depends on patients’ specific value orientations regarding health outcomes and costs. Whether viewed from a micro-level (or individual) or macro-level (or collective) perspective, healthcare services and their reforms must first be evaluated based on incremental value. Nevertheless, a primary question arises: Should the evaluation standard rely solely on the incremental gains of macro-level value or the public interest? Or should it evaluate the incremental gains of macro-level value or the public interest based on a careful balance between macro-level and micro-level values, as well as the public interest and individual values?

Second, stock value. This means that healthcare services do not produce a value-added effect for patients, leaving their overall value status unchanged. This dimension requires analysis from the “subjective-objective relationship.” From the objective perspective, many healthcare services do not actively contribute to disease treatment or improving health outcomes, yet they typically do not harm the patient’s health status either. A common example includes routine prevention and physical examinations that detect no abnormalities. Patients frequently associate such outcomes with their financial outlays, leading them to perceive these services as over-treatment, which is not necessarily the case. As scholars note, “in medicine, there is an everlasting tension between preventing the worst outcome and avoiding too many expensive unnecessary tests” (35). From the subjective perspective, stock value manifests as a form of utility. When this objective stock value is viewed through the lens of subjective utility, it transitions from a stock value into an incremental value. For instance, a physical examination that reveals no health issues does not imply a lack of value. On the contrary, individuals undergo screening because they desire to understand their actual health status. The test results satisfy this need and therefore deliver clear utility. Compared to the state of uncertainty or limited knowledge prior to the examination, the results evidently elevate the patient’s utility level. However, if identical or similar examinations are performed repeatedly in succession with normal results, the utility level will decline, demonstrating a clear trend of diminishing marginal utility.

## 3 Empirical analysis

### 3.1 Questionnaire design and survey implementation

The questionnaire was designed primarily using Likert scales. However, considering the number of items and completion time, the most critical and complex dimension—whether VBHC prioritizes health outcomes or costs—was designed as a multiple-choice question comprising three items. The options for health outcomes were drawn from Michael Porter’s three-tier health outcome measurement model. The remaining dimensions included 20 items, resulting in a 23-item questionnaire. Control variables consisted of gender, age, educational background, professional status, living or working location (primarily urban vs. county/rural areas), and geographic region (eastern, central, and western regions).

The survey was administered online, distributing questionnaires through healthcare institutions in Guangxi (including provincial-level, Nanning, and Guilin hospitals) and academic organizations like the County-level Integrated Healthcare Consortium Branch of the China Health Management Association and the Humanities and Management Science Branch of the China Association of Chinese Medicine. Of the 729 responses collected, 105 were excluded due to short completion times (<2 min) or uniform responses, leaving 624 valid questionnaires (85.60% response rate). Sample demographics showed a female majority (69.1% vs. 30.9% male), primarily aged 20–50 (20–30: 31.6%; 40–50: 29.0%; 30–40: 27.1%). Regarding education, 60.1% held bachelor’s degrees and 24.4% postgraduate degrees. Respondent roles spanned institutional staff (77.8% total: 36.2% nurses, 25.5% physicians, 10.3% executives/managers, 5.8% others), patients (3.8%), and the general public (9.3%). Lastly, 69.2% lived or worked in county-level areas (27.6% urban), with 55.9% located in western, 20.7% in eastern, and 23.4% in central China.

The questionnaire demonstrated good reliability, with a Cronbach’s alpha coefficient of 0.791. Regarding validity, content validity was ensured by rigorous theoretical analysis and feedback from 10 healthcare experts who refined the items’ content and wording. Factor analysis of the 20 items indicated strong construct validity: the KMO value was 0.839, and Bartlett’s test of sphericity yielded a chi-square value of 3390.236 (significant at the 1% level), explaining 56.596% of the variance across a clear four-factor structure. All statistical analyses, including descriptive statistics, analysis of variance (ANOVA), and factor analysis, were performed using SPSS version 20.0. The full version of the questionnaire is provided in the Appendix) for transparency.

### 3.2 Analysis of survey results

#### 3.2.1 Descriptive statistics

Descriptive statistics for the 20 Likert-scale items revealed that respondents favored equity and public interest in the efficiency-equity trade-off, yet exhibited markedly weak cost awareness when assessing healthcare value. Next, frequency analysis of the three categorical items showed: (1) Regarding treatment selection, 43.3% balanced health outcomes and costs, 30.4% sought the optimal health outcomes with minimal costs, and only 18.6% prioritized health outcomes. (2) Perspectives on considering treatment outcomes were dispersed, primarily focusing on patients’ stage-specific needs, followed by functional recovery. (3) Conversely, views on treatment costs demonstrated striking consensus, with 86.7% prioritizing the patient’s financial burden.

#### 3.2.2 ANOVA and *post-hoc* comparisons

Levene’s test for homogeneity of variance revealed that gender, educational background, and geographic region violated this assumption for only a few items. In contrast, age, living or working location, and professional status exhibited variance heterogeneity across multiple items. Items violating the assumption were analyzed using nonparametric tests or corrected procedures, whereas those satisfying it were analyzed using one-way ANOVA.

For items showing statistically significant group differences, the least significant difference (LSD) procedure was used for *post-hoc* pairwise comparisons. Table 1 reports these results, displaying only items with significant differences in perceptions. For items violating the homogeneity-of-variance assumption, nonparametric tests revealed no significant gender differences. For other demographic variables exhibiting significant effects, *post-hoc* pairwise comparisons yielded conclusions consistent with those from the ANOVA-based analyses. Accordingly, the following discussion focuses on the ANOVA results. Throughout the analysis, lower mean scores indicate stronger agreement.

1. Perceptions of the public welfare orientation versus public interest, and incremental versus stock value, differed significantly across age groups. Table 1 shows these significant differences occurred exclusively for Items 6 and 19. Item 6 (“The ‘patient’ in healthcare services is an individual whose health needs should be fulfilled as much as possible, without considering other factors”), designed around the public welfare orientation, measures efficiency and equity values. Item 19 (“If funds are invested but health outcomes remain unchanged, the healthcare service has no value”) measures incremental value. Notably, respondents aged over 60 demonstrated the highest agreement (i.e., lowest mean scores of 1.91 and 2.00, respectively), scoring significantly lower than the 40–50 and 20–30 age cohorts. Furthermore, agreement with Item 19 increased with age. This suggests that, compared to other groups, older adults lean more toward the public welfare orientation, efficiency, and incremental value.
2. Perceptions of patient-centeredness, short- and long-term reform benefits, subjective utility, and marginal value differed significantly between postgraduates and other educational cohorts. Postgraduates demonstrated the highest agreement (lowest mean scores of 1.487 and 1.618, respectively) with Item 2 (“Healthcare services only realize the interests of hospitals, medical staff, pharmaceutical companies, and the government by satisfying patient needs”) and Item 18 (“I disapprove of reform plans that solve immediate national difficulties, e.g., reducing costs, but harm long-term healthcare development”). These scores were significantly lower than those of undergraduates and junior college cohorts. This indicates that more highly educated groups more strongly endorse VBHC’s patient-centered benefit transmission mechanism and view reforms from a macro-level, long-term perspective. Conversely, postgraduates showed the lowest agreement (mean scores of 2.033 and 2.211, significantly higher than those of undergraduate and junior college cohorts) with Item 20 (“Healthcare services are valuable if they raise health awareness—i.e., subjective utility—even without improving physical health”) and Item 21 (“If continued financial input increases health outcomes but the marginal increment diminishes, the service remains valuable”). This suggests highly educated individuals focus more on objective clinical efficacy, attribute lower value to subjective utility, and are less receptive to healthcare services with diminishing marginal value.
3. Perceptions of over-treatment and the short- and long-term benefits of healthcare reform differed significantly across professional statuses. Table 1 shows these differences occurred exclusively for Items 15 and 18. For Item 15 (“Healthcare services should prioritize basic health needs, such as acute care, to avoid excessive demands like unattainable functional recovery”), pharmaceutical personnel demonstrated the lowest agreement (i.e., highest mean score of 4.0), differing significantly from all other groups, which showed no significant variance among themselves. For Item 18 (detailed above), the pharmaceutical group again exhibited the lowest agreement (mean score of 3.0), differing significantly from other professional statuses. Within the healthcare system, nurses showed significantly lower agreement (mean score of 1.903) than physicians and other cohorts. Furthermore, agreement with Item 18 correlated positively with administrative rank; healthcare institution leaders demonstrated the highest agreement (lowest mean score of 1.375), whereas non-leadership groups scored notably lower.
4. Perceptions of patient-centeredness, subjective utility, and marginal value differed significantly between urban cohorts and both township and county cohorts. Table 1 shows urban respondents differed significantly from the latter two groups across Items 2, 20, 21, and 23, while no significant variance existed between township and county respondents. For Item 2 (detailed above), the urban and county groups scored 1.529 and 1.762, respectively (*p* = 0.004). This suggests urban residents lean toward understanding “patient-centeredness” from a stakeholder perspective. Conversely, for Item 20 (detailed above), the urban cohort exhibited lower agreement (mean score of 2.105), scoring significantly higher than the township and county groups (*p* = 0.038 and *p* < 0.001, respectively). This demonstrates urban residents emphasize objective health outcomes, whereas township and county cohorts more strongly endorse subjective utility, embracing a more inclusive VBHC connotation. Regarding marginal value—measured by Item 21 (detailed above) and Item 23 (“Once health improves, if further financial input yields unchanged health outcomes, serving only a maintenance role, the service remains valuable”)—urban respondents again showed lower agreement (mean scores of 2.215 and 2.366). These scores were significantly higher than those of township and county groups (*p* = 0.038 and *p* = 0.033 for Item 21; *p* = 0.017 and *p* < 0.001 for Item 23). This indicates township and county populations are more receptive to healthcare services with diminishing marginal value.
5. Perceptions of the public welfare orientation versus public interest, equity versus efficiency, short- and long-term reform benefits, and subjective utility differed significantly across the Eastern, Central, and Western regions. Table 1 indicates significant regional variance across Items 6, 8, 14, 16, 18, and 20. Items 6 (detailed above), 8 (“The ‘patient’ is a collective; fulfilling individual needs requires considering impacts on others”), 14 (“Healthcare services should prioritize individual health needs without considering other impacts”), and 16 (“When individual needs conflict with social equity, a balance must be struck”) address whether the “patient” is viewed as an individual or a collective, exploring the relationships between the public welfare orientation and public interest, as well as the equity-efficiency tradeoff. For Items 6 and 14, Eastern respondents showed significantly higher agreement (lower mean scores of 2.845 and 3.070, respectively) than Western respondents (3.350 and 3.415; *p* < 0.001, *p* = 0.013). This indicates Eastern populations emphasize individual patients, the public welfare orientation, and efficiency. Conversely, for Items 8 and 16, Central respondents showed significantly higher agreement (mean scores of 1.493 and 1.589) than Western respondents (1.676 and 1.754; *p* = 0.014, *p* = 0.021). This indicates Central populations emphasize collective patients while favoring a balanced approach. Briefly, Eastern populations prioritize individual patients, the public welfare orientation, and efficiency, whereas Western populations prioritize collective patients, the public interest, and equity, and Central populations seek to reconcile the two, embodying a uniquely moderate value paradigm. For Item 18 (detailed above), Central respondents demonstrated significantly higher agreement than Eastern respondents (mean scores: 1.623 vs. 1.868; *p* = 0.011), valuing long-term healthcare development. Finally, for Item 20 (detailed above), Central respondents exhibited the highest agreement, differing significantly from both Eastern and Western cohorts (both *p* = 0.002). This highlights the Central population’s greater acceptance of subjective utility, reinforcing their inclusive, moderate value paradigm.

**Table 1:** Items with significant perceptual differences and their comparison groups.

Variables	Items	(I) Group	(J) Group	(I) Mean	(J) Mean	(I−J) Mean difference	Std	*p*-value
Age	B6 (4.077, 0.001^**^)	2	4	3.081	3.448	−0.366	0.133	0.006^**^
		2	6	3.081	1.909	1.172	0.401	0.004^**^
		3	6	3.213	1.909	1.304	0.403	0.001^**^
		4	5	3.448	3.031	0.416	0.188	0.027^*^
		4	6	3.448	1.909	1.538	0.402	0.000^**^
	E19 (4.19, 0.001^**^)	1	6	4	2	2	0.975	0.041^*^
		2	4	2.939	2.564	0.376	0.131	0.004^**^
		2	5	2.939	2.297	0.642	0.183	0.000^**^
		2	6	2.939	2	0.939	0.393	0.017^*^
		3	5	2.704	2.297	0.407	0.186	0.029^*^
Educational attainment	A2 (5.047, 0.002^**^)	2	3	1.585	1.795	−0.21	0.102	0.040^*^
		3	4	1.795	1.487	0.308	0.085	0.000^**^
	D18 (2.765, 0.041^*^)	2	4	1.915	1.618	0.296	0.104	0.004^**^
	E20 (4.97, 0.002^**^)	2	4	1.798	2.033	−0.235	0.106	0.028^*^
		3	4	1.733	2.033	−0.3	0.078	0.000^**^
	E21 (2.713, 0.044^*^)	3	4	2.021	2.211	−0.189	0.086	0.027^*^
Respondent identity	D15 (1.909, 0.026^*^)	1	12	1.542	4	−2.458	0.629	0.000^**^
		2	12	1.704	4	−2.296	0.608	0.000^**^
		3	12	1.85	4	−2.15	0.607	0.000^**^
		4	12	1.875	4	−2.125	0.675	0.002^**^
		5	12	1.857	4	−2.143	0.615	0.001^**^
		6	12	1.722	4	−2.278	0.621	0.000^**^
		9	12	1.5	4	−2.5	0.854	0.004^**^
		10	12	2	4	−2	0.74	0.007^**^
		12	13	4	1.429	2.571	0.685	0.000^**^
		12	14	4	1.8	2.2	0.619	0.000^**^
		12	16	4	1.931	2.069	0.614	0.001^**^
	D18 (2.359, 0.004^**^)	1	12	1.667	3	−1.333	0.575	0.021^*^
		2	13	1.673	1.903	−0.23	0.081	0.005^**^
		2	12	1.673	3	−1.327	0.556	0.017^*^
		3	5	1.903	1.5	0.403	0.117	0.001^**^
		3	12	1.903	3	−1.097	0.555	0.049^*^
		3	13	1.903	1.286	0.617	0.3	0.040^*^
		4	12	1.375	3	−1.625	0.618	0.009^**^
		5	12	1.5	3	−1.5	0.563	0.008^**^
		6	12	1.722	3	−1.278	0.568	0.025^*^
		12	13	3	1.286	1.714	0.627	0.006^**^
		12	14	3	1.65	1.35	0.566	0.017^*^
		12	16	3	1.69	1.31	0.562	0.020^*^
Place of work or residence	A2 (3.562, 0.014^*^)	2	3	1.762	1.529	0.233	0.08	0.004^**^
	E20 (10.378, 0.000^**^)	1	3	1.643	2.105	−0.462	0.223	0.038^*^
		2	3	1.706	2.105	−0.399	0.072	0.000^**^
	E21 (3.245, 0.022^*^)	1	3	1.571	2.215	−0.644	0.247	0.009^**^
		2	3	2.044	2.215	−0.171	0.08	0.033^*^
	E23 (6.403, 0.000^**^)	1	3	1.714	2.366	−0.652	0.272	0.017^*^
		2	3	2.016	2.366	−0.35	0.088	0.000^**^
	B6 (7.292, 0.001^**^)	1	3	2.845	3.35	−0.505	0.134	0.000^**^
	B8 (3.084, 0.046^*^)	2	3	1.493	1.676	−0.183	0.074	0.014^*^
	D14 (3.209, 0.041^*^)	1	3	3.07	3.415	−0.346	0.138	0.013^*^
	D16 (3.143, 0.044^*^)	2	3	1.589	1.754	−0.165	0.071	0.021^*^
	D18 (3.299, 0.038^*^)	1	2	1.868	1.623	0.245	0.095	0.011^*^
	E20 (6.015, 0.003^**^)	1	2	1.922	1.616	0.306	0.098	0.002^**^
		2	3	1.616	1.86	−0.243	0.08	0.002^**^

This table reports only those chi-square test results for which group differences are statistically significant. Cell counts and category percentages are omitted for brevity. ^*^Indicates significance at the 5% level, and ^**^indicates significance at the 1% level.

#### 3.2.3 Chi-square tests

Chi-square tests for the three categorical items (Items 10–12) revealed no significant differences regarding gender or geographic region. Table 2 reports only the statistically significant results.

1. Older adults overwhelmingly prioritize health outcomes over other age cohorts. When evaluating these outcomes, survival duration is secondary; instead, middle-aged and older cohorts focus heavily on functional recovery. However, regarding treatment costs, all groups unanimously prioritize the patient’s financial burden.
  Table 2 shows significant age-related differences for Item 10 (“Choosing a medical treatment option”; *χ*^2^ = 41.912, *p* = 0.003). Specifically, 58.33% of respondents over 60 prioritized health outcomes, far exceeding other cohorts (20–25%). Conversely, balancing health outcomes and costs was favored by the 30–40 and 40–50 age groups (44.67 and 43.98%, respectively), but selected by zero respondents over 60. While seeking optimal health outcomes at minimal cost was not the top overall preference, it was chosen by 42.65% of 50–60-year-olds and 41.67% of those over 60, higher than other groups (20–30%). This suggests that preference for health outcomes increases with age, while younger populations increasingly balance outcomes with costs—though no group prioritizes costs outright. This aligns perfectly with the earlier ANOVA results, likely because younger cohorts are more sensitive to economic burden, whereas older adults prioritize health outcomes (i.e., disease burden).
  Similarly, responses to Item 11 (“Considering treatment outcomes”) differed significantly by age (*χ*^2^ = 67.384, *p* < 0.001). The 50–60 and >60 age groups focused predominantly on functional recovery (45.31 and 45.45%, respectively), greatly exceeding other cohorts (20–30%). Additionally, 27.27% of the >60 group prioritized survival duration, compared to <15% for other groups. Conversely, choices among respondents under 40 were more dispersed, demonstrating varying attention to dimensions like disease recurrence or recovery sustainability.
2. The postgraduate group prioritizes health outcomes, whereas other educational cohorts tend to favor a balanced approach between health outcomes and costs. Furthermore, the junior college and postgraduate groups prioritize the degree of functional recovery at rates significantly higher than the undergraduate group. However, none of these groups prioritize cost above all else.
  Table 2 indicates that the educational background variable shows significant differences regarding Item 10, “Choosing a medical treatment option” (*χ*^2^ = 24.225, *p* = 0.019). Specifically, 30.26% of the postgraduate group prioritizes health outcomes, while the proportions for all other educational groups remain below 17%. Regarding the option to “comprehensively consider health outcomes and costs to seek a balance,” the selection rates for the junior college, undergraduate, and postgraduate groups were 43.62, 46.67, and 34.21%, respectively. Meanwhile, strategies such as “considering the option with the best health outcomes and the lowest cost” or simply “treatment cost” were not prioritized options. Overall, all educational cohorts lean toward balancing costs and health outcomes, with the undergraduate group exhibiting this tendency most prominently.
  Similarly, significant differences exist among educational groups regarding Item 11, “Considering treatment outcomes” (*χ*^2^ = 29.712, *p* = 0.040). Specifically, 34.04% of the junior college group and 31.58% of the postgraduate group selected “the degree of functional recovery,” compared to 23.73% for the undergraduate group. In addition, 32.80% of the undergraduate group and 26.32% of the postgraduate group would choose outcomes based on the patient’s primary needs during a specific period, whereas this proportion was 17.02% for the junior college group. Conversely, minimal attention was paid to the patient’s survival duration, and even less consideration was given to whether the treatment might cause new health issues or to the length of the treatment period. Overall, the educational background variable reflects a pragmatic orientation; however, the postgraduate group demonstrates a certain degree of idealism in comparison.
3. The general public and patients prioritize health outcomes, whereas other groups—particularly healthcare institution personnel such as physicians—tend to seek a balanced approach between health outcomes and costs. Notably, no group prioritizes costs outright. However, when considering specific types of costs, leaders of healthcare institutions lean toward prioritizing hospital costs, whereas all other groups prioritize the patient’s financial burden.
  Table 2 indicates that groups with different professional statuses exhibit significant differences in their responses to Item 10 and Item 12 (*χ*^2^ = 80.359, *p* = 0.007; *χ*^2^ = 79.550, *p* = 0.008). Regarding Item 10, “Choosing a medical treatment option,” the general public and patients prioritize health outcomes, with selection rates of 41.38 and 33.33%, respectively. These proportions are significantly higher than those of other groups. Furthermore, “considering the option with the best health outcomes and the lowest costs” is not the most preferred choice overall. On this point, patients, the general public, and healthcare institution staff demonstrate a relatively high degree of consistency (ranging between 27 and 37%). When it comes to seeking a balanced approach between health outcomes and costs, physicians and staff from health administrative departments show very high selection rates (all exceeding 50%). Conversely, patients and the general public exhibit the lowest rates (around 25%). Almost no group prioritizes treatment costs as their primary consideration.
  Regarding Item 12, “Considering treatment costs,” with the exception of healthcare institution leaders (37.5%), all other identity groups exhibit extremely high proportions (all above 70%) prioritizing the patient’s financial burden. However, 50.0% of healthcare institution leaders, 28.57% of party and government leaders, and 25% of general staff in health administrative departments tend to prioritize hospital costs. Additionally, 12.5% of healthcare institution leaders prioritize the burden on the medical insurance fund, an option rarely prioritized by any other identity group.
4. Urban populations prioritize health outcomes, whereas county and township populations factor in costs more heavily, leaning toward a balanced approach between health outcomes and costs. All groups tend to prioritize the restoration of patient function. However, the urban population pursues a higher level of health outcomes compared to their county and township counterparts. Specifically, urban residents care deeply about the patient’s survival duration, township residents are highly concerned with the side effects of treatment, and county and township populations place great importance on the likelihood of disease recurrence and the sustainability of recovery.
  Table 2 indicates significant differences among the urban, county, and township groups regarding Item 10 and Item 11 (*χ*^2^ = 33.047, *p* < 0.001; *χ*^2^ = 36.589, *p* = 0.006). Regarding Item 10, “Choosing a medical treatment option,” 30.23% of the urban population prioritizes health outcomes, a rate significantly higher than that of the county (14.35%) and township (7.14%) populations. When it comes to prioritizing the option with the best health outcomes and the lowest costs, there is little difference among the urban, county, and township groups, with selection rates of 31.40, 30.09, and 28.57%, respectively. However, regarding the choice to “comprehensively consider health outcomes and costs to seek a balance,” the differences among the three are highly significant. The proportion for the township group reaches 57.14%, whereas the rates for the county and urban groups are 47.69 and 31.40%, respectively.
  Regarding Item 11, “Considering treatment outcomes,” 16.86% of the urban population prioritizes the patient’s survival duration, which is significantly higher than those of the county (9.49%) and township (0%) populations. In contrast, there is little difference among the three groups when prioritizing the degree of functional recovery or choosing outcomes based on the patient’s primary needs during a specific period. However, when prioritizing the side effects and complications of treatment, the township group’s rate (21.43%) is significantly higher than that of the urban (7.56%) and county (7.18%) groups. Finally, regarding the likelihood of disease recurrence and the sustainability of recovery, the urban population’s selection rate (13.95%) is significantly lower than that of the township (28.57%) and county (24.77%) populations.

**Table 2:** Results of cross-tabulation and chi-square tests.

Variables	Items	*χ* ^2^	*p*-value
Age	10. When choosing a medical treatment option, what would you prioritize?	44.012	0.001**
	11. When considering treatment outcomes, which outcome should be prioritized?	119.394	0.000**
Educational attainment	10. When choosing a medical treatment option, what would you prioritize?	24.225	0.019*
	11. When considering treatment outcomes, which outcome should be prioritized?	29.712	0.04*
Respondent identity	10. When choosing a medical treatment option, what would you prioritize?	80.359	0.007**
	12. When considering treatment costs, which cost should be prioritized?	79.550	0.008**
Place of work or residence	10. When choosing a medical treatment option, what would you prioritize?	33.047	0.001**
	11. When considering treatment outcomes, which outcome should be prioritized?	36.589	0.006**

This table reports statistically significant results from the cross-tabulation and chi-square analyses only. For brevity, cell counts, and the corresponding category percentages are not reported. ^*^Indicates significance at the 5% level, and ^**^Indicates significance at the 1% level.

## 4 Conclusions and policy recommendations

### 4.1 Discussion

First, regarding the relationships between the individual and collective, public welfare orientation and public interest, and efficiency and equity, older adults and eastern populations lean toward the individual, public welfare orientation, and efficiency, whereas other groups favor the collective, equity, and the public interest. Regarding whether healthcare value should be measured by costs, despite substantial divergence, respondents demonstrate weak cost awareness. When choosing treatment options, respondents do not prioritize health outcomes or costs individually, favoring a balanced approach, followed by options offering the best health outcomes at the lowest cost. Regarding specific outcomes, opinions are somewhat divergent; however, functional recovery remains the primary priority, whereas survival duration—the highest-level outcome—is not prioritized. Conversely, regarding specific costs, there is a strong consensus on prioritizing patients’ financial burdens.

Second, gender has no substantial impact on the perception of VBHC connotations. However, regarding healthcare reforms, men are significantly more concerned about the long-term sustainability of the healthcare system than women.

Third, the age variable significantly influences the perception of VBHC connotations. Generally, young and middle-aged cohorts lean toward the public interest and equity, whereas older adults prioritize efficiency (i.e., fulfilling their own health needs) and the public welfare orientation; furthermore, younger cohorts possess stronger cost awareness. As age increases, individuals increasingly emphasize health outcomes—particularly objective health outcomes—valuing quality of life over survival duration. Conversely, younger cohorts focus on the likelihood of disease recurrence and recovery sustainability. This divergence may occur because young and middle-aged groups bear heavy financial burdens and possess strong cost awareness, aiming to reinforce equity to alleviate economic strain, whereas older adults face heavy disease burdens and prioritize health outcomes to maximize quality of life and lifespan. Additionally, this focus on functional recovery may reflect older adults’ high demand for independent living amid the growing trend of living alone.

Fourth, the educational attainment variable has a limited overall impact on the perception of VBHC connotations. However, highly educated groups lean toward equity, viewing healthcare reforms from macro and long-term perspectives while pursuing objective health outcomes and adhering firmly to the patient-centered principle. This may be because populations with broader knowledge and stronger theoretical thinking capacities analyze issues more deeply and grasp policy intentions more accurately.

Fifth, the respondent identity variable has a crucial and highly complex impact on the perception of VBHC connotations. The public primarily understands “patient-centeredness” strictly from patient needs, whereas physicians view it through a dual lens, considering both patient needs and stakeholder interests. Leaders tend to view issues through their official roles, allowing them to consider healthcare reform from macro and long-term perspectives while emphasizing costs; for instance, healthcare institution leaders focus heavily on hospital costs, which likely explains why current policy design primarily targets cost control. In contrast, regular healthcare staff focus on their responsibilities and prioritize health outcomes, while the public views issues directly through the lens of personal interests, placing them between leaders and staff. Patients emphasize efficiency, leaders (e.g., healthcare institution leaders) lean toward social equity, and physicians and nurses occupy a middle ground. Furthermore, unlike medical staff who acknowledge the subjective utility of healthcare services, patients and the public are less receptive to it. Health administrative personnel more readily accept diminishing marginal value, whereas the public finds it unacceptable, with patients and medical staff occupying a middle ground. When selecting treatment options, patients and the public prioritize health outcomes, whereas medical staff (e.g., physicians) favor a balanced approach between health outcomes and costs. Conversely, health administrative personnel distinctly focus on costs and favor balanced solutions. These complex variations likely stem from how personal interests shape perceptions of VBHC connotations. Professional responsibilities and official roles are inextricably linked to these interests; consequently, individuals favor healthcare services that align with their positions.

Sixth, urban–rural disparities substantially impact the perception of VBHC connotations due to differences in exposure, ideologies, and income. Compared with the narrower perspective of county and township residents, urban residents are more inclined to understand “patient-centeredness” from a multi-stakeholder perspective and emphasize objective health outcomes. In contrast, county and township residents are more receptive to subjective utility and diminishing marginal value. Urban residents prioritize health outcomes and survival duration, whereas county and township residents consider costs more heavily, leaning toward a balanced approach and emphasizing the likelihood of disease recurrence and recovery sustainability. Ultimately, this indicates that urban residents pursue a higher tier of health outcomes than their rural counterparts. This divergence may stem from urban residents’ broader exposure and more complex ideologies, which enable them to understand “patient-centeredness” from multi-stakeholder perspectives; moreover, their higher incomes incline them toward objective health outcomes and incremental value, whereas the opposite holds true for county and township residents.

Seventh, regional economic development is a crucial factor influencing the perception of VBHC connotations. Eastern populations emphasize individual patients and efficiency, whereas western populations focus on collective patients and equity; meanwhile, central populations lean toward collective patients but attempt to reconcile equity and efficiency, embodying a unique, moderate value paradigm. Furthermore, compared to eastern populations, central populations place greater importance on the long-term development of healthcare reform and exhibit higher acceptance and inclusiveness toward subjective utility than both eastern and western populations. These differences may be deeply tied to regional economic development. In economically developed regions, populations possess stronger cost awareness and tend to measure healthcare value through costs, whereas those in less developed regions place less emphasis on these costs.

## 5 Conclusion

Based on the empirical analysis, this study yields several core conclusions:

1. Overall, respondents demonstrate weak cost awareness. They do not prioritize health outcomes or costs individually, favoring a balanced approach. Furthermore, they prioritize patients’ financial burdens and favor functional recovery over survival duration.
2. Older adults and eastern populations lean toward the individual, the public welfare orientation, and efficiency. In contrast, other age groups and western populations favor the collective, equity, and the public interest. Meanwhile, central populations focus on the collective patient perspective but attempt to reconcile equity and efficiency, embodying a unique, moderate value paradigm.
3. Urban residents tend to understand “patient-centeredness” through a multi-stakeholder lens, emphasizing objective health outcomes and survival duration. In contrast, county and township residents are more receptive to subjective utility and diminishing marginal value, considering costs more frequently.
4. Leaders tend to view issues through their official roles and emphasize costs, whereas regular healthcare staff focus on their responsibilities and prioritize health outcomes. Patients emphasize efficiency, leaders lean toward social equity, and physicians and nurses occupy a middle ground. Unlike medical staff, patients and the public are less receptive to the subjective utility of healthcare services and find diminishing marginal value unacceptable. When selecting treatment options, patients and the public prioritize health outcomes, whereas medical staff favor a balanced approach between health outcomes and costs.

### 5.1 Policy recommendations

1. Healthcare system reforms and policy formulation should still generally align with the public’s values of equity and the public interest, while continuing to reinforce the public welfare orientation of the healthcare system.
2. Cost awareness should be strengthened to measure healthcare value through costs; however, this must be premised on prioritizing health outcomes. Cost reduction is merely a means rather than an end, with the ultimate goal being to improve health outcomes. Therefore, cost awareness education should be enhanced among the general public—particularly older adults, urban residents, and populations in the western region. Conversely, leaders, especially party and government officials, must adopt a health outcomes-first mindset.
3. Healthcare services should prioritize restoring patients’ physical and mental functions over extending survival duration. This not only aligns with patients’ health needs but also carries strong social significance, resonating with the concept of healthy aging.
4. Urban and rural healthcare institutions require differentiated positioning. Urban healthcare institutions, particularly tertiary hospitals, must prioritize health outcomes, emphasizing objective health outcomes and incremental value. In contrast, county and township healthcare institutions should achieve an appropriate balance between health outcomes and costs, as well as between objective health outcomes and subjective utility (e.g., patient experience).
5. Furthermore, healthcare institutions across geographical regions require differentiated strategic positioning. Specifically, healthcare institutions in the eastern region must strengthen technological innovation and continuously improve objective health outcomes. In contrast, those in the central and especially western regions should focus heavily on improving the equity and accessibility of healthcare services. They must achieve an appropriate balance between objective health outcomes and subjective utility. Moreover, these regions should rely on state support to absorb and integrate technological innovations from the eastern region.

### 5.2 Limitations and future prospects

While our sample is concentrated in healthcare institutions, which was a necessary approach to ensure respondents’ familiarity with the specialized concept of VBHC, the structural imbalance, particularly the limited representation of the general public, constitutes a limitation of this study. Although our findings offer valuable exploratory insights, the generalizability of these results to the broader population should be interpreted with caution. Future research should prioritize expanding the sample size across more diverse groups to further validate our conclusions.

## Data Availability

The original contributions presented in the study are included in the article/Supplementary material, further inquiries can be directed to the corresponding author.
