# Supplementary material for: The connotations of Value-Based Healthcare: interpretive perspectives and differences in perceptions
Source: Front Public Health. 2026 Jun 17;14:1866891. doi: 10.3389/fpubh.2026.1866891 (PMC13318970; doi:10.3389/fpubh.2026.1866891)
Supplement: Supplementary file 1 [file Data_Sheet_1.DOCX]

Note: The red numbers in parentheses (1, 2, 3, 4, etc.) correspond to the option codes used in Wenjuanxing data analysis and are included to identify each option during data processing.

Questionnaire on the Connotations of Value-Based Healthcare

Dear Madam/Sir,

Hello. We are conducting a research project on Value-Based Healthcare (VBHC), with the aim of informing China’s healthcare-system reform. Your response is very important to us. Thank you for taking the time to complete this questionnaire. This survey is anonymous. The information you provide will be used only for academic research, and all collected data will be kept strictly confidential. Please feel free to answer the questions.

I. Basic Information

1. Your gender:

A. Male (1) B. Female (2)

2. Your age:

A. Under 20 years old (excluding 20) (1) B. 20-30 years old (excluding 30) (2) C. 30-40 years old (excluding 40) (3) D. 40-50 years old (excluding 50) (4) E. 50-60 years old (excluding 60) (5) F. 60 years old and above (6)

3. Your educational attainment:

A. Secondary school or below (1) B. Junior college (2) C. Bachelor's degree (3) D. Graduate degree (4)

4. Your respondent role (please select the category closest to your current role):

A. Patient (including former patients) (1)

B. Physician (including medical interns) (2)

C. Nurse (including nursing interns) (3)

D. Leader of a healthcare institution (4)

E. Middle manager in a healthcare institution (5)

F. Other healthcare-institution staff (including other interns) (6)

G. Leader in a healthcare security department (7)

H. Staff member in a medical insurance/healthcare security department (8)

I. Leader in a health supervisory authority (9)

J. Staff member in a health supervisory authority (10)

K. Leader in a pharmaceutical firm (11)

L. Staff member in a pharmaceutical firm (12)

M. Party/government leader (13)

N. Staff member of a medical college or university (14)

O. Village doctor (15)

P. Member of the general public (not included in the categories above) (16)

5. Your place of work or residence:

A. Township (1) B. County seat (including county-level city) (2) C. Urban area (3) D. Rural area (4)

6. The region where you work or live:

A. Eastern China (1) B. Central China (2) C. Western China (3)

II. Questionnaire Items

Please judge each statement based on your own understanding and tick the corresponding option. In this questionnaire, "health outcomes" or "healthcare outcomes" refer to overall outcomes after considering treatment side effects and other related factors.

(I) The Relational Category of VBHC: Subject-Object Relations or Subjective-Objective Relations?

1. Healthcare services involve multiple actors, including patients, health professionals, hospitals, the public, pharmaceutical firms, and governments; however, to have value, healthcare services must place patients at the center.

A. Strongly agree (1) B. Somewhat agree (2) C. Not sure (3) D. Somewhat disagree (4) E. Strongly disagree (5)

2. Only when healthcare services meet patients' needs can the interests of hospitals, health professionals, pharmaceutical firms, governments, and other actors be realized.

A. Strongly agree (1) B. Somewhat agree (2) C. Not sure (3) D. Somewhat disagree (4) E. Strongly disagree (5)

3. The value of healthcare services lies in their ability to improve patients' health.

A. Strongly agree (1) B. Somewhat agree (2) C. Not sure (3) D. Somewhat disagree (4) E. Strongly disagree (5)

4. The value of healthcare services lies in patients measuring the degree of health improvement against input costs and judging whether the service is worth the cost.

A. Strongly agree (1) B. Somewhat agree (2) C. Not sure (3) D. Somewhat disagree (4) E. Strongly disagree (5)

5. Different patients perceive the importance of costs differently; therefore, their judgments about the value of healthcare services also differ.

A. Strongly agree (1) B. Somewhat agree (2) C. Not sure (3) D. Somewhat disagree (4) E. Strongly disagree (5)

(II) The Patient in Patient-Centered Care: Individual or Population?

6. In healthcare services, the "patient" is an individual; their health needs should be met as fully as possible, without considering other factors.

A. Strongly agree (1) B. Somewhat agree (2) C. Not sure (3) D. Somewhat disagree (4) E. Strongly disagree (5)

7. In healthcare services, the "patient" is an individual; however, meeting their health needs must be premised on not undermining the public interest.

A. Strongly agree (1) B. Somewhat agree (2) C. Not sure (3) D. Somewhat disagree (4) E. Strongly disagree (5)

8. In healthcare services, the "patient" is a population; when meeting an individual patient's health needs, one should consider whether this affects other patients.

A. Strongly agree (1) B. Somewhat agree (2) C. Not sure (3) D. Somewhat disagree (4) E. Strongly disagree (5)

9. In healthcare services, the "patient" may be either an individual or a population; the health needs of both individuals and populations should be considered comprehensively.

A. Strongly agree (1) B. Somewhat agree (2) C. Not sure (3) D. Somewhat disagree (4) E. Strongly disagree (5)

(III) The VBHC Formula: Outcome Primacy or Cost Primacy?

10. When choosing a medical treatment plan, you would:

A. Prioritize health outcomes; only when health outcomes are the same or similar would you consider treatment costs (1)

B. Prioritize treatment costs; only when treatment costs are the same or similar would you consider health outcomes (2)

C. Choose the option that simultaneously achieves the best outcomes and the lowest cost (3)

D. Consider outcomes and costs jointly and seek a balanced option (4)

E. Choose a plan based on the patient's predominant needs at a given stage (5)

11. When considering health outcomes, which outcome should be prioritized?

A. Patients' survival duration (1)

B. Degree of patients' functional recovery (2)

C. Treatment duration (3)

D. Treatment side effects and complications (4)

E. The likelihood of disease recurrence or sustainability of recovery (5)

F. Whether treatment might cause new health problems (6)

G. Select the outcome based on the patient's predominant needs at a given stage (7)

12. When considering treatment costs, which cost should be prioritized?

A. Hospital costs (1)

B. Patients' financial burden (2)

C. Patients' non-economic costs, such as time (3)

D. Burden on the medical insurance fund (4)

E. Deductions from health professionals’ performance pay (5)

(IV) The Scope of VBHC: Micro-Level or Macro-Level?

13. Healthcare services should prioritize equity and avoid meeting the health needs of individual patients in ways that adversely affect other patients or the public.

A. Strongly agree (1) B. Somewhat agree (2) C. Not sure (3) D. Somewhat disagree (4) E. Strongly disagree (5)

14. Healthcare services should prioritize meeting patients' health needs as quickly as possible, without considering other impacts.

A. Strongly agree (1) B. Somewhat agree (2) C. Not sure (3) D. Somewhat disagree (4) E. Strongly disagree (5)

15. Healthcare services should prioritize patients' health needs, but only basic needs (e.g., emergency treatment for acute diseases), avoiding excessive health demands (e.g., demanding functional restoration to a level that is difficult to achieve).

A. Strongly agree (1) B. Somewhat agree (2) C. Not sure (3) D. Somewhat disagree (4) E. Strongly disagree (5)

16. When an individual patient's treatment needs conflict with social equity, one should consider the value of both and seek a balance.

A. Strongly agree (1) B. Somewhat agree (2) C. Not sure (3) D. Somewhat disagree (4) E. Strongly disagree (5)

17. I do not support practices in which an individual healthcare-service component creates value for patients but the overall value is not evident (e.g., when side effects or complications make the overall value unclear).

A. Strongly agree (1) B. Somewhat agree (2) C. Not sure (3) D. Somewhat disagree (4) E. Strongly disagree (5)

18. I do not support healthcare reform plans that address current national difficulties (e.g., reducing costs) but are detrimental to healthcare development in the long run.

A. Strongly agree (1) B. Somewhat agree (2) C. Not sure (3) D. Somewhat disagree (4) E. Strongly disagree (5)

(V) The Nature of Value in VBHC: Incremental or Stock?

19. If financial resources are invested but health outcomes do not change, then the healthcare service has no value.

A. Strongly agree (1) B. Somewhat agree (2) C. Not sure (3) D. Somewhat disagree (4) E. Strongly disagree (5)

20. Even if healthcare services do not improve physical health, they are still valuable if they improve patients' health awareness (i.e., subjective utility).

A. Strongly agree (1) B. Somewhat agree (2) C. Not sure (3) D. Somewhat disagree (4) E. Strongly disagree (5)

21. If additional financial input increases health outcomes, but the magnitude of outcome gains keeps diminishing, the healthcare service is still valuable.

A. Strongly agree (1) B. Somewhat agree (2) C. Not sure (3) D. Somewhat disagree (4) E. Strongly disagree (5)

22. If health outcomes continue to improve as financial input keeps increasing, but the estimated gain in outcomes is smaller than the additional financial input, then the healthcare service has no value.

A. Strongly agree (1) B. Somewhat agree (2) C. Not sure (3) D. Somewhat disagree (4) E. Strongly disagree (5)

23. After health improves, if additional financial input does not change health outcomes (serving only a maintenance function), the healthcare service is still valuable.

A. Strongly agree (1) B. Somewhat agree (2) C. Not sure (3) D. Somewhat disagree (4) E. Strongly disagree (5)
