# Supplementary material for: The connotations of Value-Based Healthcare: interpretive perspectives and differences in perceptions
Source: Front Public Health. 2026 Jun 17;14:1866891. doi: 10.3389/fpubh.2026.1866891 (PMC13318970; doi:10.3389/fpubh.2026.1866891)
Supplement: Supplementary file 2 [file Table_1.DOCX]

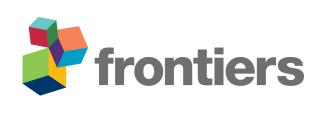


***Supplementary Material***

The statistical analyses reported in this supplementary material, including descriptive statistics, analysis of variance (ANOVA), and factor analysis, were completed using SPSS 20.0. To enhance the transparency and reproducibility of the study, the original system-generated analytical tables, figures, and related explanations are reported below.

**Supplementary Table 1. Sample characteristics of the questionnaire survey**

This table presents the demographic characteristics and structural composition of the 624 valid responses in detail. The data show that respondents affiliated with healthcare institutions (including health professionals and managers) accounted for the largest share, at 77.8%; patients and members of the general public accounted for 3.8% and 9.3%, respectively; and the regional distribution was dominated by western China (55.9%). This key variable information objectively reflects the data source of the study and provides the basis for subsequent analyses of perceptual differences across groups.

**Table 1. Sample characteristics of the questionnaire survey**

| **Variable** | **Category** | **Frequency (n)** | **Percentage (%)** |
| --- | --- | --- | --- |
| Gender | Male | 193 | 30.9 |
|  | Female | 431 | 69.1 |
|  | Total | 624 | 100.0 |
| Age | Under 20 years old (excluding 20) | 2 | 0.3 |
|  | 20-30 years old (excluding 30) | 197 | 31.6 |
|  | 30-40 years old (excluding 40) | 169 | 27.1 |
|  | 40-50 years old (excluding 50) | 181 | 29.0 |
|  | 50-60 years old (excluding 60) | 64 | 10.3 |
|  | 60 years old and above | 11 | 1.8 |
|  | Total | 624 | 100.0 |
| Educational attainment | Secondary school or below | 3 | 0.5 |
|  | Junior college | 94 | 15.1 |
|  | Bachelor's degree | 375 | 60.1 |
|  | Graduate degree | 152 | 24.4 |
|  | Total | 624 | 100.0 |
| Respondent role | Patient (including former patients) | 24 | 3.8 |
|  | Physician (including medical interns) | 159 | 25.5 |
|  | Nurse (including nursing interns) | 226 | 36.2 |
|  | Leader of a healthcare institution | 8 | 1.3 |
|  | Middle manager in a healthcare institution | 56 | 9.0 |
|  | Other healthcare-institution staff (including other interns) | 36 | 5.8 |
|  | Staff member in a healthcare security department | 1 | 0.2 |
|  | Leader in a health supervisory authority | 2 | 0.3 |
|  | Staff member in a health supervisory authority | 4 | 0.6 |
|  | Leader in a pharmaceutical firm | 1 | 0.2 |
|  | Staff member in a pharmaceutical firm | 2 | 0.3 |
|  | Party/government leader | 7 | 1.1 |
|  | Staff member of a medical college or university | 40 | 6.4 |
|  | Member of the general public (not included in the categories above) | 58 | 9.3 |
|  | Total | 624 | 100.0 |
| Place of work or residence | Township | 14 | 2.2 |
|  | County seat (including county-level city) | 432 | 69.2 |
|  | Urban area | 172 | 27.6 |
|  | Rural area | 6 | 1.0 |
|  | Total | 624 | 100.0 |
| Region of work or residence | Eastern China | 129 | 20.7 |
|  | Central China | 146 | 23.4 |
|  | Western China | 349 | 55.9 |
|  | Total | 624 | 100.0 |

**Supplementary Table 2. Descriptive statistics (N = 624)**

To comprehensively present the overall distributional characteristics of the scale data, this study analyzed the central tendency and dispersion of all Likert-scale items. The results indicate that respondents generally leaned toward equity and the public interest when considering the relationship between efficiency and equity. However, views were somewhat dispersed on whether the value of healthcare services should be measured in cost terms, and the overall pattern showed relatively weak cost awareness.

**Table 2. Descriptive statistics of the questionnaire**

| **Item** | **Mean** | **Median** | **Mode** | **Standard deviation** | **Minimum** | **Maximum** |
| --- | --- | --- | --- | --- | --- | --- |
| A1. Healthcare services involve multiple actors, including patients, health professionals, hospitals, the public, pharmaceutical firms, and governments; however, to have value, healthcare services must place patients at the center. | 1.381 | 1 | 1 | 0.6197 | 1 | 5 |
| A2. Only when healthcare services meet patients' needs can the interests of hospitals, health professionals, pharmaceutical firms, governments, and other actors be realized. | 1.686 | 1 | 1 | 0.8909 | 1 | 5 |
| A3. The value of healthcare services lies in their ability to improve patients' health. | 1.418 | 1 | 1 | 0.6678 | 1 | 5 |
| A4. The value of healthcare services lies in patients measuring the degree of health improvement against input costs and judging whether the service is worth the cost. | 2.061 | 2 | 1 | 1.1623 | 1 | 5 |
| A5. Different patients perceive the importance of costs differently; therefore, their judgments about the value of healthcare services also differ. | 1.554 | 1 | 1 | 0.6895 | 1 | 5 |
| B6. In healthcare services, the "patient" is an individual; their health needs should be met as fully as possible, without considering other factors. | 3.197 | 3 | 4 | 1.3098 | 1 | 5 |
| B7. In healthcare services, the "patient" is an individual; however, meeting their health needs must be premised on not undermining the public interest. | 1.542 | 1 | 1 | 0.7441 | 1 | 5 |
| B8. In healthcare services, the "patient" is a population; when meeting an individual patient's health needs, one should consider whether this affects other patients. | 1.617 | 2 | 1 | 0.7575 | 1 | 5 |
| B9. In healthcare services, the "patient" may be either an individual or a population; the health needs of both individuals and populations should be considered comprehensively. | 1.458 | 1 | 1 | 0.6472 | 1 | 5 |
| D13. Healthcare services should prioritize equity and avoid meeting the health needs of individual patients in ways that affect other patients or the public. | 1.55 | 1 | 1 | 0.7283 | 1 | 5 |
| D14. Healthcare services should prioritize meeting patients' health needs as quickly as possible, without considering other impacts. | 3.311 | 4 | 4 | 1.3442 | 1 | 5 |
| D15. Healthcare services should prioritize patients' health needs, but only basic needs (e.g., emergency treatment for acute diseases), avoiding excessive health demands (e.g., demanding functional restoration to a level that is difficult to achieve). | 1.801 | 2 | 2 | 0.8624 | 1 | 5 |
| D16. When an individual patient's treatment needs conflict with social equity, one should consider the value of both and seek a balance. | 1.691 | 2 | 2 | 0.7248 | 1 | 5 |
| D17. I do not support practices in which an individual healthcare-service component creates value for patients but the overall value is not evident (e.g., because of side effects or complications). | 1.737 | 2 | 2 | 0.7611 | 1 | 5 |
| D18. I do not support healthcare reform plans that address current national difficulties (e.g., reducing costs) but are detrimental to healthcare development in the long run. | 1.742 | 2 | 1 | 0.7927 | 1 | 5 |
| E19. If financial resources are invested but health outcomes do not change, then the healthcare service has no value. | 2.688 | 3 | 2 | 1.2846 | 1 | 5 |
| E20. Even if healthcare services do not improve physical health, they are still valuable if they improve patients' health awareness (i.e., subjective utility). | 1.816 | 2 | 2 | 0.8189 | 1 | 5 |
| E21. If additional financial input increases health outcomes, but the magnitude of outcome gains keeps diminishing, the healthcare service is still valuable. | 2.079 | 2 | 2 | 0.8933 | 1 | 5 |
| E22. If health outcomes continue to improve as financial input keeps increasing, but the estimated magnitude of outcome gains is smaller than the magnitude of the increase in financial input, then the healthcare service has no value. | 3.09 | 3 | 4 | 1.229 | 1 | 5 |
| E23. After health improves, if additional financial input does not change health outcomes (serving only a maintenance function), the healthcare service is still valuable. | 2.103 | 2 | 2 | 0.9923 | 1 | 5 |

**Supplementary Table 3. Homogeneity-of-variance test results (Levene's test)**

Before conducting analysis of variance (ANOVA) to examine perceptual differences across groups, this study used Levene's test to diagnose the homogeneity-of-variance assumption for each demographic and categorical variable. This procedure determined whether subsequent analyses should directly apply one-way ANOVA or instead use corresponding nonparametric tests or corrected procedures for specific items with unequal variances.

**Table 3. Items with unequal variances (Levene's test)**

| **Variable** | **Item** | **F** | **p** |
| --- | --- | --- | --- |
| Gender | B8. | 8.384 | 0.004** |
|  | D14. | 4.191 | 0.041* |
|  | E20. | 5.133 | 0.024* |
| Age | A1. | 5.231 | 0.000** |
|  | A2. | 2.457 | 0.032* |
|  | A5. | 2.649 | 0.022* |
|  | D14. | 3.789 | 0.002** |
|  | E21. | 2.597 | 0.025* |
|  | E23. | 2.928 | 0.013* |
| Educational attainment | A1. | 5.938 | 0.001** |
|  | A3. | 4.137 | 0.006** |
|  | B6. | 2.843 | 0.037* |
|  | D13. | 3.736 | 0.011* |
| Respondent role | A1. | 3.745 | 0.000** |
|  | A2. | 2.510 | 0.002** |
|  | A3. | 2.383 | 0.004** |
|  | A4. | 2.141 | 0.011* |
|  | A5. | 2.099 | 0.013* |
|  | B6. | 2.478 | 0.003** |
|  | B8. | 1.782 | 0.042* |
|  | D13. | 1.818 | 0.037* |
|  | D14. | 2.612 | 0.002** |
|  | E20. | 2.179 | 0.009** |
|  | E23. | 1.975 | 0.021* |
| Place of work or residence | A1. | 12.951 | 0.000** |
|  | A3. | 8.342 | 0.000** |
|  | A4. | 3.495 | 0.015* |
|  | B6. | 3.567 | 0.014* |
|  | B9. | 5.469 | 0.001** |
|  | D13. | 3.579 | 0.014* |
| Region of work or residence | A3. | 4.735 | 0.009** |
|  | A4. | 3.448 | 0.032* |
|  | B9. | 3.247 | 0.040* |

Notes: 1. * p < 0.05; ** p < 0.01. 2. To simplify the table, group-specific data for each variable were omitted.

**Supplementary Table 4. KMO and Bartlett's test of sphericity results**

To assess whether the sample data were suitable for exploratory factor analysis, this study conducted the KMO test and Bartlett's test of sphericity. The results show that the KMO value was 0.839 and that Bartlett's test of sphericity was significant at the 1% level, indicating that the sample data had a sound basis for construct validity and were highly suitable for factor reduction analysis.

Table 4. KMO and Bartlett's test

| **Test** | **Statistic** | **Value** |
| --- | --- | --- |
| Kaiser-Meyer-Olkin Measure of Sampling Adequacy |  | .839 |
| Bartlett's Test of Sphericity | Approx. Chi-Square | 3390.236 |
|  | df | 153 |
|  | Sig. | .000 |

**Supplementary Table 5. Item communalities**

During factor extraction, communalities measure the extent to which each item is explained by the extracted factors. After two items with relatively low communalities were removed, exploratory factor analysis of the retained 18 Likert-scale items indicated satisfactory construct validity. The communalities of all retained items reached acceptable levels, with no extremely low values observed, further supporting the construct validity of the scale.

**Table 5. Communalities**

| **Item** | **Initial** | **Extraction** |
| --- | --- | --- |
| A1. Healthcare services involve multiple actors, including patients, health professionals, hospitals, the public, pharmaceutical firms, and governments; however, to have value, healthcare services must place patients at the center. | 1.000 | .487 |
| A2. Only when healthcare services meet patients' needs can the interests of hospitals, health professionals, pharmaceutical firms, governments, and other actors be realized. | 1.000 | .550 |
| A3. The value of healthcare services lies in their ability to improve patients' health. | 1.000 | .535 |
| A4. The value of healthcare services lies in patients measuring the degree of health improvement against input costs and judging whether the service is worth the cost. | 1.000 | .479 |
| B6. In healthcare services, the "patient" is an individual; their health needs should be met as fully as possible, without considering other factors. | 1.000 | .639 |
| B7. In healthcare services, the "patient" is an individual; however, meeting their health needs must be premised on not undermining the public interest. | 1.000 | .475 |
| B8. In healthcare services, the "patient" is a population; when meeting an individual patient's health needs, one should consider whether this affects other patients. | 1.000 | .524 |
| B9. In healthcare services, the "patient" may be either an individual or a population; the health needs of both individuals and populations should be considered comprehensively. | 1.000 | .483 |
| D13. Healthcare services should prioritize equity and avoid meeting the health needs of individual patients in ways that affect other patients or the public. | 1.000 | .541 |
| D14. Healthcare services should prioritize meeting patients' health needs as quickly as possible, without considering other impacts. | 1.000 | .656 |
| D16. When an individual patient's treatment needs conflict with social equity, one should consider the value of both and seek a balance. | 1.000 | .519 |
| D17. I do not support practices in which an individual healthcare-service component creates value for patients but the overall value is not evident (e.g., because of side effects or complications). | 1.000 | .691 |
| D18. I do not support healthcare reform plans that address current national difficulties (e.g., reducing costs) but are detrimental to healthcare development in the long run. | 1.000 | .675 |
| E19. If financial resources are invested but health outcomes do not change, then the healthcare service has no value. | 1.000 | .618 |
| E20. Even if healthcare services do not improve physical health, they are still valuable if they improve patients' health awareness (i.e., subjective utility). | 1.000 | .555 |
| E21. If additional financial input increases health outcomes, but the magnitude of outcome gains keeps diminishing, the healthcare service is still valuable. | 1.000 | .561 |
| E22. If health outcomes continue to improve as financial input keeps increasing, but the estimated magnitude of outcome gains is smaller than the magnitude of the increase in financial input, then the healthcare service has no value. | 1.000 | .602 |
| E23. After health improves, if additional financial input does not change health outcomes (serving only a maintenance function), the healthcare service is still valuable. | 1.000 | .598 |

Extraction Method: Principal Component Analysis.

**Supplementary Table 6. Total variance explained**

This study used principal component analysis to extract initial factors with eigenvalues greater than 1. The results show that the four major factors ultimately extracted jointly explained 56.596% of the total variance. The factor extraction ratio was appropriate and could adequately cover the main information contained in the original questionnaire.

**Table 6. Total variance explained**

| **Component** | **Initial Eigenvalues** |  |  | **Extraction Sums of Squared Loadings** |  |  | **Rotation Sums of Squared Loadings** |  |  |
| --- | --- | --- | --- | --- | --- | --- | --- | --- | --- |
|  | **Total** | **% of Variance** | **Cumulative %** | **Total** | **% of Variance** | **Cumulative %** | **Total** | **% of Variance** | **Cumulative %** |
| 1 | 4.819 | 26.774 | 26.774 | 4.819 | 26.774 | 26.774 | 3.318 | 18.434 | 18.434 |
| 2 | 2.287 | 12.703 | 39.477 | 2.287 | 12.703 | 39.477 | 2.552 | 14.177 | 32.611 |
| 3 | 1.646 | 9.146 | 48.623 | 1.646 | 9.146 | 48.623 | 2.466 | 13.702 | 46.313 |
| 4 | 1.435 | 7.973 | 56.596 | 1.435 | 7.973 | 56.596 | 1.851 | 10.282 | 56.596 |
| 5 | .957 | 5.315 | 61.911 |  |  |  |  |  |  |
| 6 | .739 | 4.105 | 66.016 |  |  |  |  |  |  |
| 7 | .683 | 3.797 | 69.813 |  |  |  |  |  |  |
| 8 | .650 | 3.610 | 73.423 |  |  |  |  |  |  |
| 9 | .622 | 3.455 | 76.878 |  |  |  |  |  |  |
| 10 | .603 | 3.348 | 80.226 |  |  |  |  |  |  |
| 11 | .584 | 3.245 | 83.471 |  |  |  |  |  |  |
| 12 | .527 | 2.925 | 86.396 |  |  |  |  |  |  |
| 13 | .491 | 2.730 | 89.126 |  |  |  |  |  |  |
| 14 | .474 | 2.635 | 91.761 |  |  |  |  |  |  |
| 15 | .413 | 2.292 | 94.053 |  |  |  |  |  |  |
| 16 | .386 | 2.142 | 96.195 |  |  |  |  |  |  |
| 17 | .365 | 2.027 | 98.222 |  |  |  |  |  |  |
| 18 | .320 | 1.778 | 100.000 |  |  |  |  |  |  |

Extraction Method: Principal Component Analysis.

**Supplementary Figure 1. Scree plot**

In addition to the eigenvalue criterion, this study also referred to the slope changes shown in the scree plot. The scree plot visually shows that the curve becomes relatively flat beginning with the fifth factor. This pattern supported the decision that extracting four core factors was the optimal factor extraction solution.


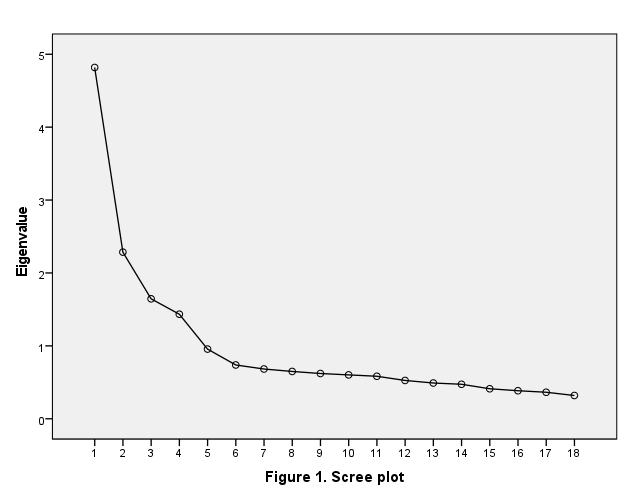


**Supplementary Table 7. Rotated factor loading matrix**

To make the substantive theoretical meaning of each factor clearer, this study rotated the factor loading matrix using the Varimax orthogonal rotation method. The rotated factor structure was clear, with no serious cross-loading. Four core dimensions were ultimately extracted: patient-centeredness, equity-efficiency, macro-micro, and incremental-stock. These results provide a solid empirical basis for constructing the assessment model for the connotations of Value-Based Healthcare (VBHC).

**Table 7. Rotated factor loading matrixa**

| **Item** | **Factor 1** | **Factor 2** | **Factor 3** | **Factor 4** |
| --- | --- | --- | --- | --- |
| A3. The value of healthcare services lies in their ability to improve patients' health. | .710 |  |  |  |
| A2. Only when healthcare services meet patients' needs can the interests of hospitals, health professionals, pharmaceutical firms, governments, and other actors be realized. | .691 |  |  |  |
| A1. Healthcare services involve multiple actors, including patients, health professionals, hospitals, the public, pharmaceutical firms, and governments; however, to have value, healthcare services must place patients at the center. | .689 |  |  |  |
| B9. In healthcare services, the "patient" may be either an individual or a population; the health needs of both individuals and populations should be considered comprehensively. | .622 |  |  |  |
| B7. In healthcare services, the "patient" is an individual; however, meeting their health needs must be premised on not undermining the public interest. | .610 |  |  |  |
| B8. In healthcare services, the "patient" is a population; when meeting an individual patient's health needs, one should consider whether this affects other patients. | .583 |  | .331 |  |
| D13. Healthcare services should prioritize equity and avoid meeting the health needs of individual patients in ways that affect other patients or the public. | .551 |  | .479 |  |
| D14. Healthcare services should prioritize meeting patients' health needs as quickly as possible, without considering other impacts. |  | .795 |  |  |
| B6. In healthcare services, the "patient" is an individual; their health needs should be met as fully as possible, without considering other factors. |  | .753 |  |  |
| E22. If health outcomes continue to improve as financial input keeps increasing, but the estimated magnitude of outcome gains is smaller than the magnitude of the increase in financial input, then the healthcare service has no value. |  | .751 |  |  |
| E19. If financial resources are invested but health outcomes do not change, then the healthcare service has no value. |  | .589 | .349 | -.365 |
| A4. The value of healthcare services lies in patients measuring the degree of health improvement against input costs and judging whether the service is worth the cost. | .479 | .493 |  |  |
| D18. I do not support healthcare reform plans that address current national difficulties (e.g., reducing costs) but are detrimental to healthcare development in the long run. |  |  | .807 |  |
| D17. I do not support practices in which an individual healthcare-service component creates value for patients but the overall value is not evident (e.g., because of side effects or complications). |  |  | .803 |  |
| D16. When an individual patient's treatment needs conflict with social equity, one should consider the value of both and seek a balance. | .336 |  | .613 |  |
| E23. After health improves, if additional financial input does not change outcomes (serving only a maintenance function), the healthcare service is still valuable. |  |  |  | .765 |
| E21. If additional financial input increases health outcomes, but the magnitude of outcome gains keeps diminishing, the healthcare service is still valuable. |  |  |  | .714 |
| E20. Even if healthcare services do not improve physical health, they are still valuable if they improve patients' health awareness (i.e., subjective utility). |  |  | .374 | .636 |

Extraction Method: Principal Component Analysis. Rotation Method: Varimax with Kaiser Normalization. a. Rotation converged in 7 iterations.
